# FABP7 drives an inflammatory response in human astrocytes and is upregulated in Alzheimer’s disease

**DOI:** 10.1007/s11357-023-00916-0

**Published:** 2023-09-09

**Authors:** Haylee L. Hamilton, Noah A. Kinscherf, Garrett Balmer, Mariana Bresque, Shahriar M. Salamat, Marcelo R. Vargas, Mariana Pehar

**Affiliations:** 1 Division of Geriatrics and Gerontology, Department of Medicine, University of Wisconsin-Madison, 600 Highland Avenue, CSC K6/447, Madison, WI 53792 USA; 2 Neuroscience Training Program, University of Wisconsin-Madison, Madison, WI USA; 3 Department of Neurology, University of Wisconsin-Madison, Madison, WI USA; 4 Department of Pathology and Laboratory Medicine, University of Wisconsin-Madison, Madison, WI USA; 5 Department of Neurological Surgery, University of Wisconsin Madison, Madison, WI USA; 6 Geriatric Research Education Clinical Center, William S. Middleton Memorial Veterans Hospital, Madison, WI USA

**Keywords:** Alzheimer’s disease, Amyloid β (Aβ), Astrocytes, Brain lipid binding protein (BLBP), Fatty acid binding protein, FABP7, Inflammation

## Abstract

Alzheimer’s disease (AD), the most common cause of dementia in the elderly, is characterized by the accumulation of intracellular neurofibrillary tangles, extracellular amyloid plaques, and neuroinflammation. In partnership with microglial cells, astrocytes are key players in the regulation of neuroinflammation. Fatty acid binding protein 7 (FABP7) belongs to a family of conserved proteins that regulate lipid metabolism, energy homeostasis, and inflammation. FABP7 expression is largely restricted to astrocytes and radial glia-like cells in the adult central nervous system. We observed that treatment of primary hippocampal astrocyte cultures with amyloid β fragment 25–35 (Aβ_25–35_) induces FABP7 upregulation. In addition, FABP7 expression is upregulated in the brain of APP/PS1 mice, a widely used AD mouse model. Co-immunostaining with specific astrocyte markers revealed increased FABP7 expression in astrocytes. Moreover, astrocytes surrounding amyloid plaques displayed increased FABP7 staining when compared to non-plaque-associated astrocytes. A similar result was obtained in the brain of AD patients. Whole transcriptome RNA sequencing analysis of human astrocytes differentiated from induced pluripotent stem cells (i-astrocytes) overexpressing FABP7 identified 500 transcripts with at least a 2-fold change in expression. Gene Ontology enrichment analysis identified (i) positive regulation of cytokine production and (ii) inflammatory response as the top two statistically significant overrepresented biological processes. We confirmed that wild-type FABP7 overexpression induces an NF-κB-driven inflammatory response in human i-astrocytes. On the other hand, the expression of a ligand-binding impaired mutant FABP7 did not induce NF-κB activation. Together, our results suggest that the upregulation of FABP7 in astrocytes could contribute to the neuroinflammation observed in AD.

**Supplementary Information:**

The online version contains supplementary material available at 10.1007/s11357-023-00916-0.

## Introduction

Alzheimer’s disease (AD), the most common cause of dementia in the elderly, is characterized by the accumulation of intracellular neurofibrillary tangles and extracellular amyloid plaques, together with neuropil threads and gliosis. These pathological changes lead to massive neuronal loss constrained to the limbic and association cortices and subcortical ascending projection systems [1–3]. Longitudinal studies indicate that in familial and sporadic cases of AD, the deposition of the amyloid β-peptide (Aβ), generated by sequential proteolysis of the amyloid precursor protein (APP), starts decades before the onset of clinical symptoms [4–7]. These pathological changes induce an inflammatory reaction that contributes to the development of AD [8–10]. The critical role of neuroinflammation in the pathology is supported by the identification of gene variants of immune receptors, such as TREM2 and CD33, which increase the risk of developing the disease [11, 12], and by epidemiologic studies showing that treatment with non-steroidal anti-inflammatory drugs (NSAIDs) lowers the risk of developing the disease [13, 14]. However, although sustained inflammation induces neurotoxicity, some components of the inflammatory response promote resolution and facilitate Aβ clearance [8, 15].

In partnership with microglia, astrocytes are key players in the regulation of neuroinflammation. They express multiple receptors involved in innate immunity, which, following activation, regulate the secretion of soluble mediators, including cytokines and chemokines, that regulate both innate and adaptive immune responses [16, 17]. Astrocytes respond to injury or disease by adopting a reactive phenotype. Both beneficial and harmful effects have been attributed to reactive astrocytes in AD. Reactive astrocytes seem to play an important role in the degradation of amyloid plaques and secrete multiple trophic factors important for neuronal survival and function [18–20]. On the other hand, by secreting proinflammatory and cytotoxic mediators, astrocytes can induce neuronal death [9, 21]. Accordingly, astrocytes treated with Aβ peptide fragment 25–35 alter tau metabolism and reduce the viability of co-cultured hippocampal neurons [22, 23].

We recently showed that upregulation of fatty acid binding protein 7 (FABP7) expression in primary spinal cord astrocyte cultures induces a proinflammatory phenotype that could contribute to the neurotoxicity observed in amyotrophic lateral sclerosis [24]. FABP7 belongs to a family of small (~15 kDa) and widely expressed intracellular proteins that were originally considered biologically silent chaperones of fatty acids. However, fatty acid binding proteins (FABPs) have now emerged as central regulators of lipid metabolism, energy homeostasis, and inflammation [25, 26]. FABPs participate in fatty acid metabolism by regulating their uptake and transport, but they also regulate signaling processes by distributing and/or sequestering substrates for enzymes and ligands for nuclear receptors [27–32]. In the adult central nervous system, FABP7 expression is largely restricted to astrocytes and radial glia-like cells [33–36]. A recent report indicates FABP7 is expressed at low levels in specific neuronal populations and that neuronal FABP7 expression is negatively regulated by the presence of apolipoprotein ε4 (*APOE4*), a gene variant that increases the risk of developing AD [37]. However, this regulatory mechanism of FABP7 expression does not seem to be active in astrocytes [37].

Previous reports have shown that FABP7 is upregulated in patients with different pathological conditions [38–41]. However, the consequences of this upregulation in the biology of astrocytes have not been elucidated. Here, we investigated the expression of FABP7 in the context of AD and evaluated the effect of FABP7 overexpression in human astrocytes differentiated from induced pluripotent stem cells (iPSCs).

## Methods

### Reagents

All chemicals and reagents were obtained from Sigma-Aldrich unless otherwise specified. Cell culture media, serum, and supplements were obtained from Life Technologies unless otherwise indicated. Primers were obtained from Integrated DNA Technologies.

### Animals

B6.Cg-Tg(APPswe,PSEN1dE9)85Dbo/Mmjax (APP/PS1) mice [42] were obtained from the Jackson Laboratory and maintained as hemizygous animals in a congenic C57BL/6J background. The genotype was determined by PCR. All animal procedures were carried out in accordance with the recommendations in the Guide for the Care and Use of Laboratory Animals of the NIH. The experimental procedures reported in this publication were approved by the Animal Care and Use Committee at the University of Wisconsin-Madison (Animal Welfare Assurance number A3368-01).

### Human post-mortem brain tissue

Paraffin-embedded autopsy brain tissue samples from 5 AD patients (mean age: 74 years; range: 60–84 years; sex: 2 females and 3 males) and 5 control subjects (mean age: 72.2 years; range: 62–82 years; sex: 3 females and 2 males) were provided by the Wisconsin Brain Donor Program (WBDP) Brain Bank of the Neuropathology Core of the Wisconsin Alzheimer Disease Research Center (grant NIH/NIA P50-AG033514). The use of human brain tissue was approved by the University of Wisconsin-Madison Institutional Review Board. All AD patient samples analyzed were from individuals with a high likelihood of AD (NIA-Reagan diagnosis), Braak stage VI, the presence of frequent neuritic plaques (CERAD score definite AD), and Thal phase 5.

### Histology and immunostaining

Mice were transcardially perfused with 0.1 M phosphate-buffered saline (PBS, pH 7.4), followed by 4% paraformaldehyde in PBS. Brains were removed and paraffin-embedded using standard techniques. Tissue sections (5 μm) were prepared using a microtome. Antigen retrieval was performed in the R-Universal Epitope Recovery Buffer (Electron Microscopy Sciences) using the 2100 Retriever heat-induced epitope recovery system (Electron Microscopy Sciences). Immunostaining was performed as we previously described [43]. The primary antibodies used were mouse anti-GFAP clone GA5 (1:200; Novus, NBP2-29415), mouse anti-ALDH1L1 clone 4A12 (1:1000; Novus, NBP2-50045), rabbit anti-FABP7 (1:100; Thermo Fisher, PA5-24949 or 1:500; EnCor, RPCA-FABP7), goat anti-COX2 (1:100; Novus Biologicals, NB100-868), goat anti-GFAP (1:250; Thermo Fisher, PA5-18598), and mouse anti-iNOS (1:200; R&D Systems, MAB9502). The secondary antibodies were (i) Alexa Fluor 488-conjugated goat anti-mouse (Thermo Fisher-Invitrogen) and Biotin-SP-conjugated AffiniPure goat anti-rabbit (Jackson ImmunoResearch Laboratories), followed by Alexa Fluor 594-conjugated streptavidin (Jackson ImmunoResearch Laboratories); (ii) Alexa Fluor 594-conjugated AffiniPure donkey anti-rabbit (Jackson ImmunoResearch Laboratories), Alexa Fluor 647-conjugated AffiniPure donkey anti-mouse (Jackson ImmunoResearch Laboratories), and Biotin-SP-conjugated AffiniPure donkey anti-goat (Jackson ImmunoResearch Laboratories) followed by Alexa Fluor 488-conjugated streptavidin (Jackson ImmunoResearch Laboratories). Nuclei were counterstained with DAPI (4′,6-diamidino-2-phenylindole dihydrochloride; Thermo Fisher-Invitrogen). Controls were performed omitting the primary antibody.

Amyloid plaque staining was performed after antigen retrieval using Amylo-Glo RTD (Biosensis). Immunostaining after amyloid plaque staining was performed using permeabilization and blocking solutions prepared in 0.1 M phosphate buffer pH 7.2. Images were captured on an Axio Observer 5 microscope (Carl Zeiss Microscopy) with identical settings for the experimental groups.

Quantification of FABP7 staining intensity in GFAP-positive (GFAP+) astrocytes in brain tissue samples from APP/PS1 mice or AD patients was performed using the National Institute of Health Fiji (ImageJ) software. For APP/PS1 mice, 4 animals per genotype and 2–4 cortical images per animal were acquired for analysis. A mask identifying GFAP+ astrocytes was created in Fiji. GFAP+ cells were defined as having an area of at least 11.5 μm^2^, concordant with GFAP staining. The mask identifying astrocytes was overlaid onto the FABP7 channel and area, and intensity measures were recorded. GFAP+ astrocytes with high FABP7 expression (FABP7+/GFAP+) were defined as those having FABP7 intensity greater than the median of that observed in non-transgenic animals. The area covered by FABP7+/GFAP+ cells was calculated by the sum of the areas of GFAP+ astrocytes with high FABP7 expression. FABP7 intensity in GFAP+ astrocytes was quantified using the raw integrated density per GFAP+ area. Both measures are presented as fold change values. Co-immunostaining of COX2 or iNOS with FABP7 and GFAP was captured using a 20 × 0.75 NA objective on a Nikon A1R-Si+ confocal microscope at the University of Wisconsin-Madison Biochemistry Optical Core. Images were acquired with identical settings at a *z*-stack step size of 1.05 μm. The quantification of COX2 and iNOS expression in GFAP+ astrocytes was conducted in the same manner as described above.

For human samples, 5 images per individual were analyzed. A mask identifying GFAP+ astrocytes was created as described above and overlaid onto the Amylo-Glo channel. Plaques were defined as Amylo-Glo staining areas greater than 5 μm in diameter. Astrocytes were manually classified as plaque-associated, when located at a distance below or equal to 50 μm from the edge of the plaque, and non-plaque-associated, when located outside these limits. To record FABP7 staining intensity, the same mask of GFAP+ astrocytes was overlaid onto the FABP7 channel.

### Western blot

Protein extracts from cultured cells or tissue samples were prepared in Tris-HCl buffer (pH 7.6) supplemented with 150 mM NaCl, 2 mM EDTA, 0.25% Nonidet P-40, 1% Triton X-100, and a complete protease inhibitor cocktail EDTA free (Roche). Cell extracts and tissue samples were sonicated in the indicated buffer and centrifuged at 4 °C for 10 min at 10,000 *g*. Protein concentration was determined using the bicinchoninic acid method (Pierce BCA protein assay; Thermo Fisher). Samples were resolved on SurePAGE™ Bis-Tris 8–16% gels (GenScript) and transferred to PVDF membranes using the eBlot L1 Fast Wet Transfer System (GenScript). Membranes were then incubated for 1 h in Tris-buffered saline (TBS), 0.1% Tween-20, and 5% bovine serum albumin, followed by overnight incubation at 4 °C with the primary antibody diluted in the same buffer. Amersham ECL horseradish peroxidase-conjugated secondary antibodies (Cytiva) diluted in the same buffer were incubated for 1 h at room temperature. Membranes were developed using the Amersham ECL Prime chemiluminescent detection system (Cytiva), and image acquisition was performed in a C-DiGit Chemiluminescence Western Blot Scanner (LI-COR Biosciences). Quantifications were performed using the Image Studio Software (LI-COR Biosciences). The following primary antibodies were used: mouse monoclonal anti-β-actin (clone AC-15) (1:10000; Sigma-Aldrich, A5441), rabbit anti-FABP7 (1:1000; Thermo Fisher, PA5-24949 or 1:500; EnCor, RPCA-FABP7), mouse anti-FABP7 clone AT1D1 (1:2000; LifeSpan BioSciences, LS-B6684), mouse monoclonal anti-GFP tag (1:2000; Proteintech, 66002-1-IG), and mouse monoclonal anti-α-Tubulin (DM1A) (1:1000; Cell Signaling, #3873).

### Cell culture and treatments

Primary astrocyte cultures were prepared from the hippocampus or cerebral cortex of 1-day-old pups, as we previously described [44]. Cultures were maintained in Dulbecco’s modified Eagle’s medium (DMEM) supplemented with fetal bovine serum (FBS; 10%), HEPES (3.6g/L), penicillin (100 IU/mL), and streptomycin (100 μg/mL). Cultures were > 99% pure as determined by glial fibrillary acidic protein (GFAP) immunostaining and displayed < 1% of IBA1-positive microglial cells. The Aβ peptide fragment 25–35 and the control peptide with reverse amino acid sequence Aβ 35–25 (Sigma-Aldrich) were prepared in water at a concentration of 1 mg/mL, and aliquots were used immediately. Treatment of confluent hippocampal astrocyte cultures with Aβ_25–35_ or control Aβ_35–25_ (10 μm) was performed in DMEM supplemented with 1% N2 supplement and HEPES, penicillin, and streptomycin as described above.

Induced pluripotent stem cell (iPSC) lines were obtained from the NINDS Human Cell and Data Repository or commercial vendors. The iPSC lines from healthy control subjects used were Line 1 (iPSC ID# FA0000011, healthy control), Line 2 (XCL1 iPSC, Stem Cell, healthy control), and Line 3 (iPSC ID# ND38555, healthy control). iPSCs were differentiated into induced neural progenitor cells (NPCs) using an embryoid body formation protocol as we previously described [45]. Astrocyte differentiation was achieved by culturing induced NPCs for 3 weeks in astrocyte differentiation media (STEMdif Astrocyte Differentiation Media, Stemcell), followed by 3 more weeks in astrocyte maturation media (STEMdif Astrocyte Maturation Media, Stemcell). Differentiation of NPCs into astrocytes was confirmed by assessing GFAP, S100B, ALDH1L1, and AQP4 gene expression. Following differentiation, iPSC-derived astrocytes (i-astrocytes) were maintained in complete astrocyte medium from ScienCell.

Confluent i-astrocyte cultures or primary mouse astrocyte cultures were transduced with adenovirus vectors at a multiplicity of infection of 50. Adenovirus expressing GFP, wild-type mouse FABP7 (NM_021272.3), or mutant mouse FABP7(3A), under the control of the CMV promoter, were custom produced by VectorBuilder using the pAV[Exp] adenovirus gene expression vector derived from the adenovirus serotype 5 (Ad5). Mutant FABP7 unable to bind fatty acids was generated by the substitution of three critical residues in the ligand binding pocket of FABP7 for alanine [R107, R127, and Y129; FABP7(3A)] [31, 46].

To determine cell survival, cultures were fixed with 4% paraformaldehyde and stained with DAPI. The number of cells per well was determined by fluorescence microscopy analysis in an IN Cell analyzer 2000 (GE Healthcare).

### RNA sequencing (RNA-seq) and data analysis

Total RNA was isolated from i-astrocyte cultures using TRI Reagent according to the manufacturer’s instructions. Quality control of the RNA samples was determined by TapeStation analysis (Agilent). RNA library preparation with rRNA depletion, sequencing reactions, and initial data analysis (trimming, mapping, and differential gene expression) were performed by Azenta Life Sciences. Short-read sequencing was performed on an Illumina® HiSeq platform using a 2 × 150 bp paired-end sequencing configuration. A comparison of gene expression levels between control and FABP7-overexpressing samples was performed using DESeq2. The Wald test was used to generate *p*-values and log2 fold changes. Genes with an adjusted *p*-value < 0.05 and an absolute log2 fold change > 1 were considered significantly regulated. The RNA-seq data was deposited in NCBI’s Gene Expression Omnibus (GEO; accession number GSE214628). Gene Ontology (GO) enrichment analysis was performed with PANTHER (protein annotation through evolutionary relationship) analysis tool version 17.0 [47] using the complete GO database (DOI: 10.5281/zenodo.5725227 Released 2020-11-01) and the complete list of identified transcripts as the reference list. Fisher’s exact test with the Benjamini–Hochberg false discovery rate (FDR) correction for multiple testing was used for statistical over-representation analysis.

### NF-κB reporter assay

Adenovirus expressing a firefly luciferase gene under the control of a synthetic minimal promoter containing tandem direct repeats of NF-κB response elements (Ad-NF-κB-Luc) or a *Renilla* luciferase gene under the control of a constitutive promoter (Ad-pRL-Luc) were obtained from Vector Biolabs. Adenovirus-mediated transductions were performed as described above at a multiplicity of infection of 10 and 3, respectively. After 72 h, cultures were transduced with adenovirus expressing GFP, FABP7, or FABP7(3A), as described above. Firefly and *Renilla* luciferase activities were consecutively assayed with the Dual-Glo luciferase system (Promega) 48 h later.

### ELISA and nitrate/nitrite assay

i-Astrocyte cultures were transduced with adenovirus expressing GFP, FABP7, or FABP7(3A), and 72 h later, the cell culture medium was changed to DMEM/F12 medium without phenol red supplemented with 1% N2 supplement (Thermo Fisher). After 72 h, CXCL10 protein levels and total nitrate and nitrite concentration in the conditioned media were assessed. CXCL10 protein levels were quantified using the Quantikine ELISA kit for human CXCL10/IP-10 from R&D Systems. Total nitrate and nitrite levels were determined using a nitrate/nitrite Fluorometric Assay Kit (Cayman).

### Real-time PCR

Total RNA was isolated from astrocyte cultures using TRI Reagent. RNA retrotranscription and real-time PCR were performed as previously described [48], using the QuantStudio 5 real-time PCR System (Thermo Fisher-Applied Biosystems). The sequence of the specific primers used is provided in Supplemental Table 1.

### Statistical analysis

Unless otherwise indicated, cell culture experiments were repeated in at least three independent primary culture preparations, and values from each independent experiment performed in duplicate or triplicate were combined for data reporting. Comparisons between the two groups were performed using an unpaired *t*-test with Welch’s correction. The D'Agostino-Pearson normality test (omnibus K2 test) or the Shapiro-Wilk test (depending on sample size) was used to evaluate if data samples were consistent with a Gaussian distribution. Data sets from the quantification of FABP7 immunostaining deviated from an ideal Gaussian distribution and therefore were analyzed using the Mann-Whitney nonparametric test. Multiple group comparisons were performed by one-way analysis of variance (ANOVA) followed by Tukey’s post-test. The Brown-Forsythe test for equal variances was used to confirm equal variances in data groups prior to performing an ordinary one-way ANOVA. The Welch version of one-way ANOVA followed by Dunnett’s T3 multiple comparisons post-test was used when the standard deviations of the data groups were different. However, in those cases, the same conclusion regarding statistical significance among groups was obtained using the Welch version or ordinary one-way ANOVA. When comparing the effects of genotype and treatments, two-way ANOVA was used, followed by Tukey’s post-test. Differences were declared statistically significant if *p* ≤ 0.05. All statistical computations were performed using Prism 9.0 (GraphPad Software).

## Results

**Fig. 1:**
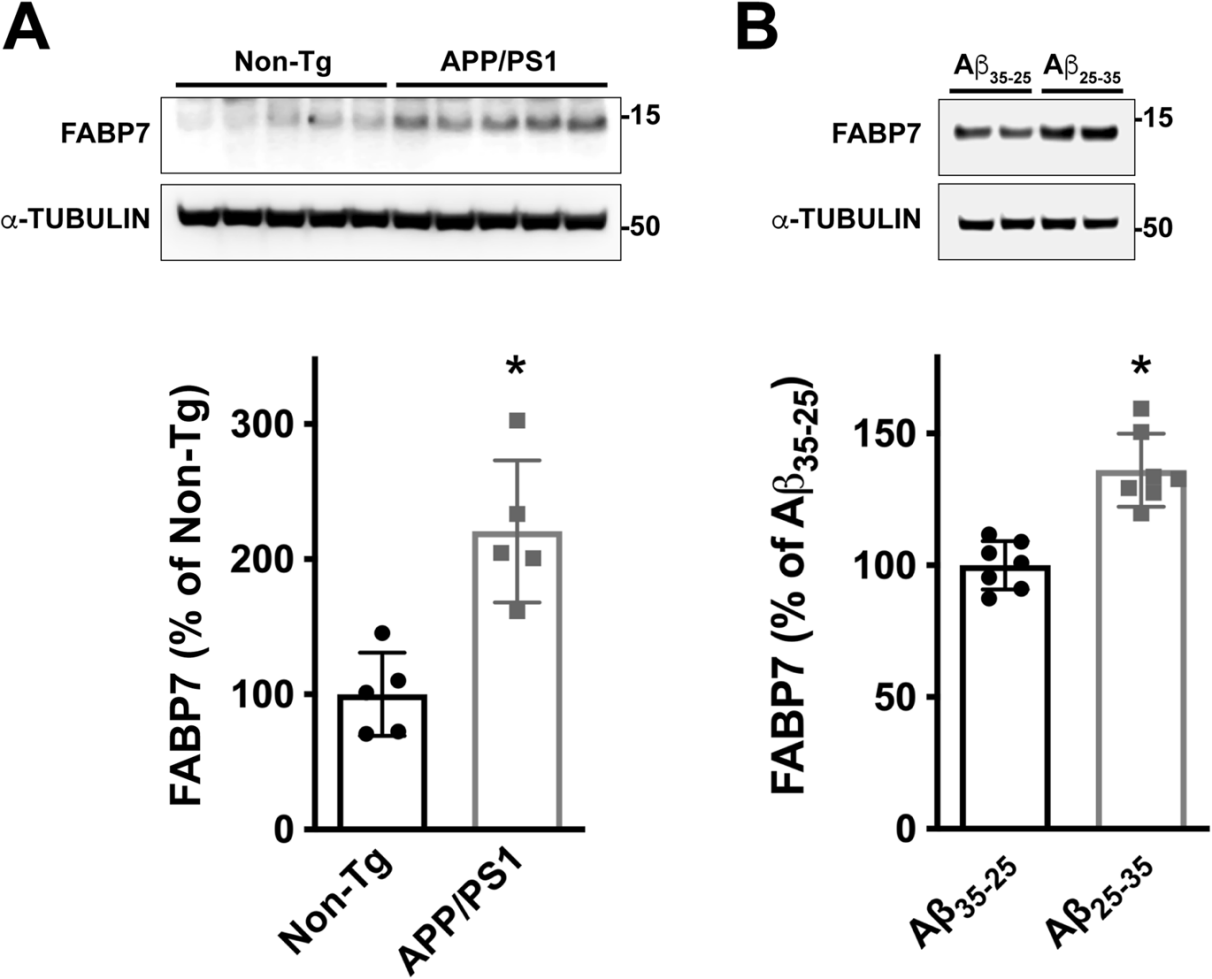
FABP7 upregulation in APP/PS1 mice and Aβ-treated astrocyte cultures. **A** Western blot analysis of FABP7 expression in the brain of 9-month-old non-transgenic (Non-Tg) or APP/PS1 mice. Each lane represents a different animal. Quantification of FABP7 protein levels is shown in the lower panel. FABP7 expression was quantified, normalized by α-TUBULIN levels, and expressed as a percentage of Non-Tg mice (mean ± SD), *p < 0.05. **B** Primary hippocampal astrocyte cultures were treated with the Aβ peptide fragment 25–35 (Aβ_25–35_; 10 μm) or the control peptide Aβ_35–25_ (10 μm), and 48 h later, FABP7 expression was analyzed by western blot. A representative western blot image is shown in the top panel, and the quantification of FABP7 protein levels is shown in the lower panel. FABP7 expression was quantified, normalized by α-TUBULIN levels, and expressed as a percentage of control Aβ_35–25_-treated cells (mean ± SD), *p < 0.05

**Fig. 2:**
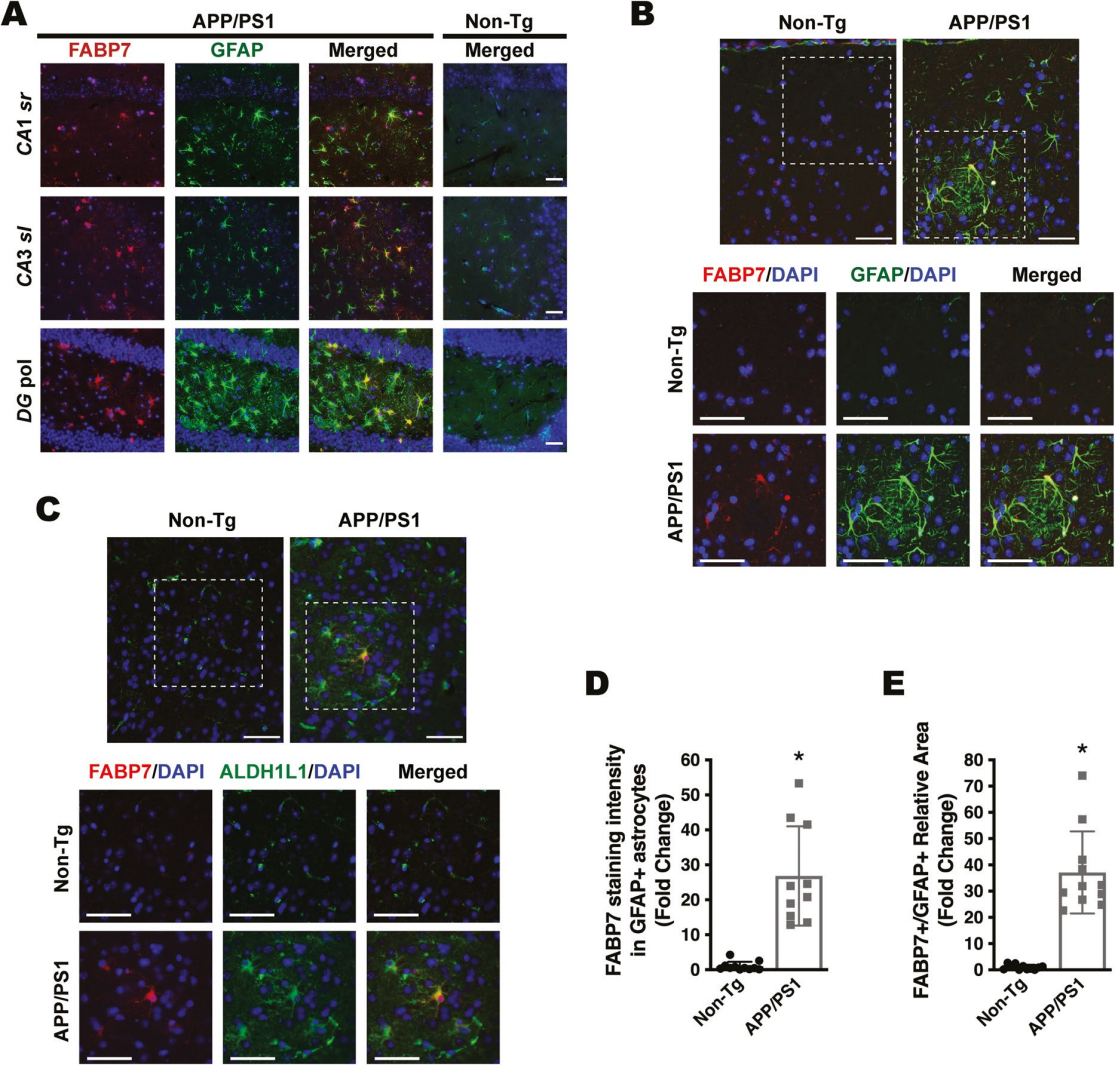
FABP7 upregulation in astrocytes of APP/PS1 mice. **A** Representative microphotographs showing FABP7 (red) and GFAP (green) immunostaining in the hippocampus of 6-month-old APP/PS1 mice and age-matched non-transgenic (Non-Tg) mice. Nuclei were counterstained with DAPI (blue). Only the merged image is shown for Non-Tg mice. DG pol., polymorphic layer of the dentate gyrus; CA3 sl, CA3 stratum lucidum. CA1 sr, CA1 stratum radiatum. Scale bar: 50 μm. **B**, **C** Co-immunostaining for FABP7 (red) and the specific astrocyte markers, GFAP (green, **B**) and ALDH1L1 (green, **C**), in the cerebral cortex of 9-month-old Non-Tg mice or APP/PS1 mice displaying widespread amyloid pathology. Nuclei were counterstained with DAPI (blue). The lower panel shows a higher magnification of the boxed area indicated in the top panel. Scale bar: 50 μm. Representative images obtained from sections where the primary antibodies were omitted are shown in Supplementary Figure 1. **D** Quantification of FABP7 staining intensity in GFAP+ astrocytes in the cerebral cortex of 9-month-old APP/PS1 and age-matched Non-Tg mice. **E** Quantification of the area occupied by GFAP+ astrocytes displaying high FABP7 expression in the cerebral cortex of 9-month-old APP/PS1 and age-matched Non-Tg mice. GFAP+ astrocytes with high FABP7 expression were defined as those having FABP7 staining intensity greater than the median of non-transgenic animals. For **D** and **E**, 2–4 images per animal were analyzed, *n* = 4 animals/genotype; data are expressed as mean ± SD, *p < 0.05

**Fig. 3:**
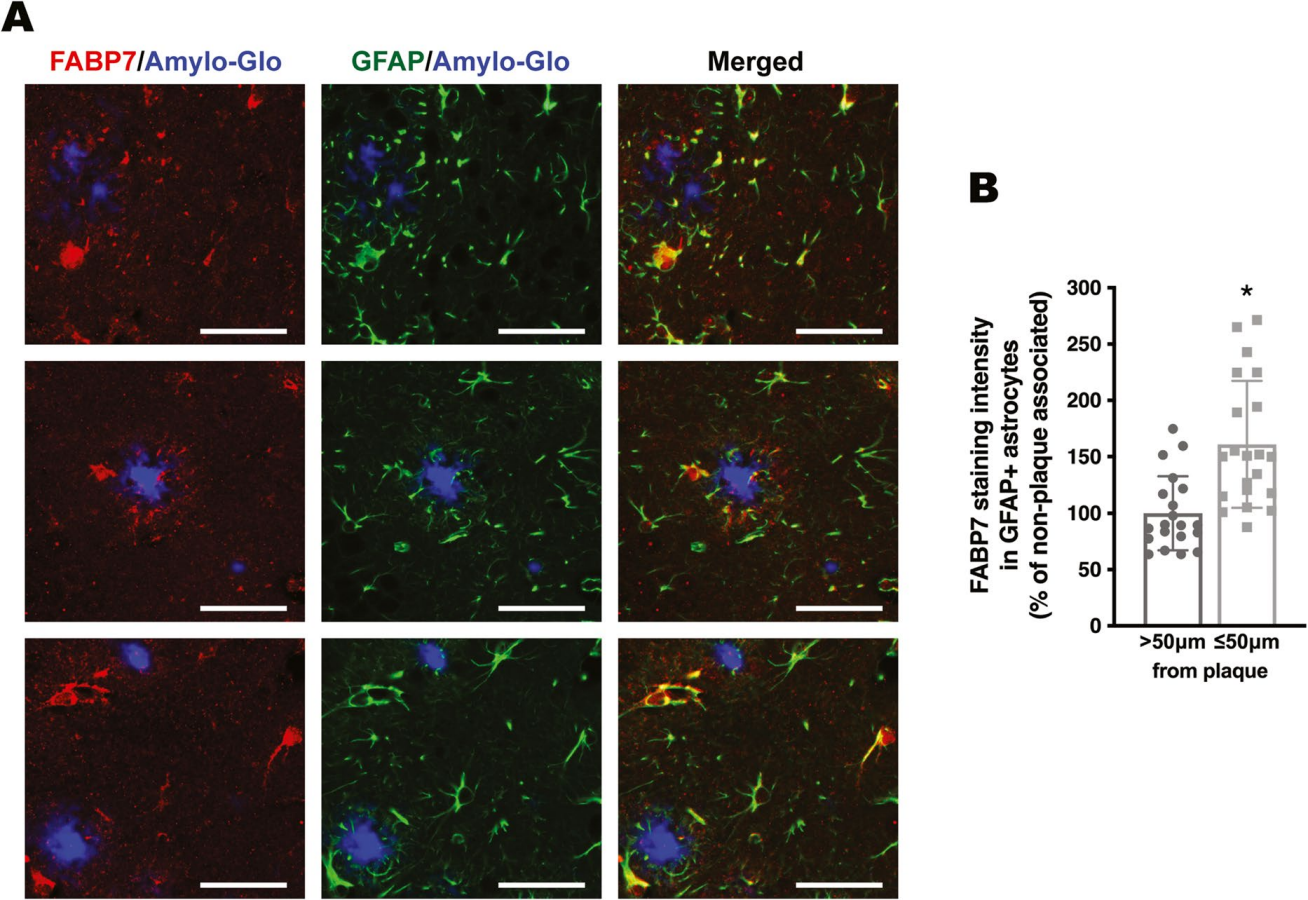
FABP7 immunostaining in astrocytes located in the vicinity of amyloid plaques. **A** Representative microphotographs showing FABP7 immunoreactive astrocytes surrounding amyloid plaques in the cerebral cortex of 9-month-old APP/PS1 mice. Amyloid plaques were stained using Amylo-Glo (blue), followed by co-immunostaining with antibodies specific for FABP7 (red) and GFAP (green). Scale bar: 50 μm. **B** Quantification of FABP7 staining intensity in GFAP+ astrocytes located within 50 μm from the edge of a plaque (plaque-associated) or beyond this boundary (non-plaque-associated; > 50 μm from the edge of a plaque). Data are expressed as a percentage of non-plaque-associated GFAP+ astrocytes. 2–4 images per animal were analyzed, *n* = 4–5 animals/genotype; data are expressed as mean ± SD, *p < 0.05

We previously showed that astrocytic FABP7 expression increases at the early stages of the disease in the spinal cord of different ALS mouse models [24]. This increase in FABP7 expression is also observed in APP/PS1 mice, a widely used AD mouse model (Fig. 1A). Western blot analysis showed an approximately 2.2-fold increase in FABP7 expression in the cerebral cortex of 9-month-old APP/PS1 mice (Fig. 1A). In addition, we observed an upregulation in FABP7 expression in primary hippocampal astrocyte cultures 48 h after treatment with the amyloid β peptide fragment Aβ_25–35_, when compared to astrocytes treated with the control reverse peptide Aβ_35–25_ (Fig. 1B). Co-immunostaining with specific astrocyte markers (GFAP and ALDH1L1) confirmed increased astrocytic FABP7 expression in the brain of APP/PS1 mice (Fig. 2). The quantification of FABP7 immunostaining revealed an increase in the intensity of FABP7 staining in GFAP+ astrocytes (Fig. 2D). In addition, we observed an increase in the area occupied by GFAP+ astrocytes displaying high FABP7 expression, defined as those having an intensity of FABP7 staining greater than the median observed in non-transgenic mice (Fig. 2E). The increase in FABP7 expression was evident in astrocytes surrounding amyloid plaques (Fig. 3). However, upregulation of FABP7 expression was not observed in all GFAP+ cells, likely reflecting the phenotypic heterogeneity of this cell type during neurodegeneration. We also observed an increase of about 60% in FABP7 staining intensity in GFAP+ astrocytes located within 50 μm from the edge of Amylo-Glo positive plaques, when compared to non-plaque-associated astrocytes (located at a distance > 50 μm from the edge of a plaque) (Fig. 3B).

**Fig. 4:**
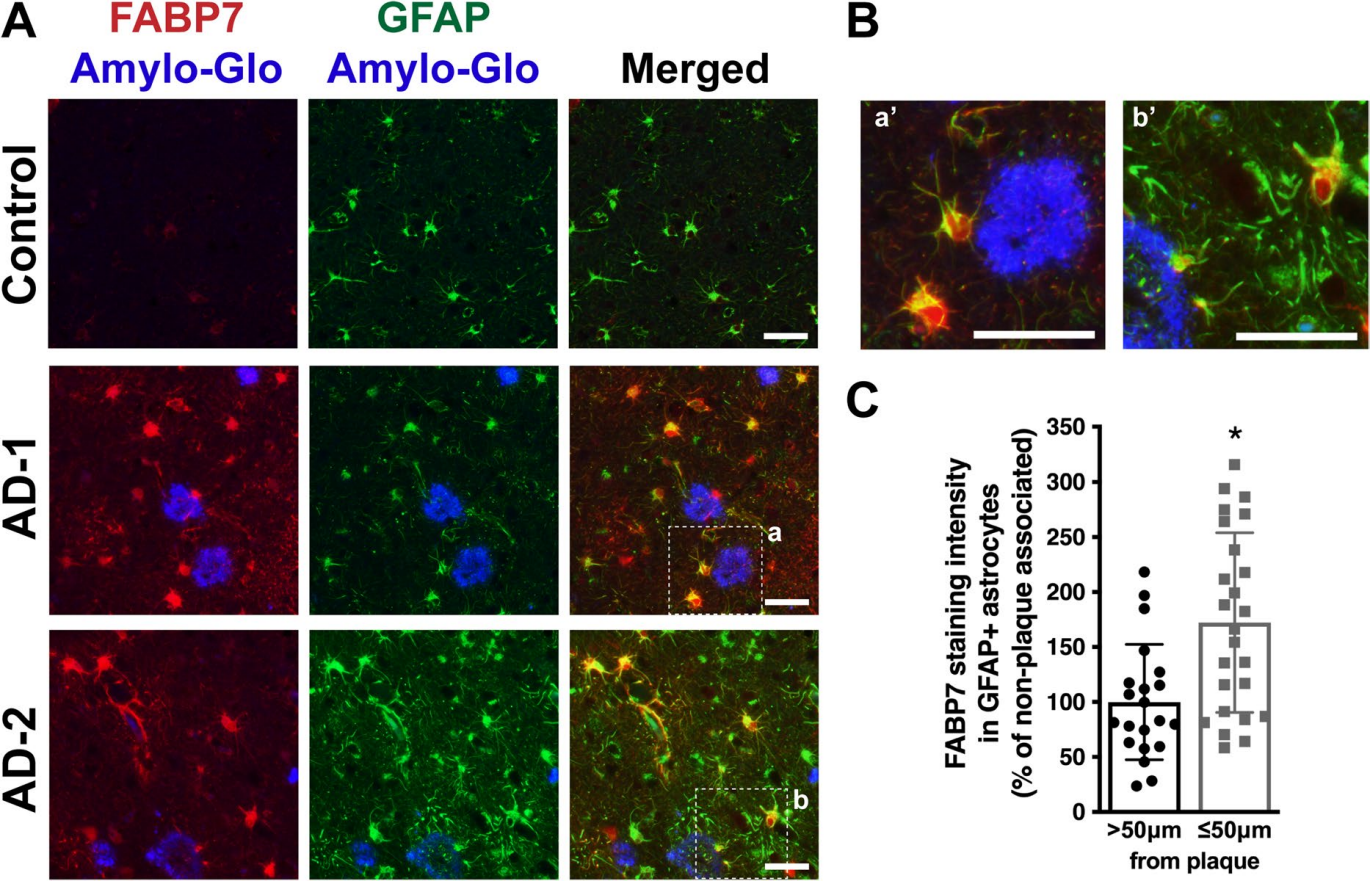
FABP7 expression in AD patients. **A** FABP7 (red) and GFAP (green) immunostaining in the middle frontal gyrus of cognitive unimpaired (CU) control and AD subjects. Amyloid plaques were stained using Amylo-Glo (blue). Representative images of one control and two different AD patients (AD-1 and AD-2) are shown. **B** Higher magnification images of the boxed areas indicated in **A**, depicting FABP7-immunoreactive astrocytes surrounding amyloid plaques. Scale bar: 50 μm. **C** Quantification of FABP7 staining intensity in GFAP+ astrocytes located within 50 μm from the edge of a plaque (plaque-associated) or beyond this boundary (non-plaque-associated; > 50 μm from the edge of a plaque). Images from 5 AD patients and 5 age-matched controls were analyzed (5 images per individual). Data are expressed as percentage of non-plaque-associated GFAP+ astrocytes, mean ± SD, *p < 0.05

It has been previously reported that FABP7 expression increases in the brains of AD patients [38]. In addition, the publicly available platform Agora, which provides proteomic data analysis from post-mortem brains of more than 500 individuals, shows evidence of FABP7 upregulation in three different brain regions of AD patients: the anterior prefrontal cortex (log2 fold change: 0.392; *Adj. p* = 0.0423), the dorsolateral prefrontal cortex (log2 fold change: 0.39; *Adj. p* = 5.76e-7), and the middle frontal gyrus (log2 fold change: 0.798; *Adj. p* = 0.0264) (data version: syn13363290-v49; https://agora.adknowledgeportal.org/genes/ENSG00000164434/evidence/protein). We demonstrate by immunostaining that FABP7 expression is observed in GFAP+ astrocytes surrounding Amylo-Glo stained plaques in the middle frontal gyrus of AD patients (Fig. 4). In addition, we observed an increase of about 70% in FABP7 staining intensity in GFAP+ astrocytes located in the vicinity of amyloid plaques (within 50 μm from the edge of a plaque) when compared to non-plaque-associated astrocytes (located at a distance > 50 μm from the edge of a plaque) (Fig. 4C). Indeed, 75.4 ± 9.3% of the area covered by plaque-associated GFAP+ astrocytes displayed high levels of FABP7 staining intensity (greater than the median observed in control patients).

**Fig. 5:**
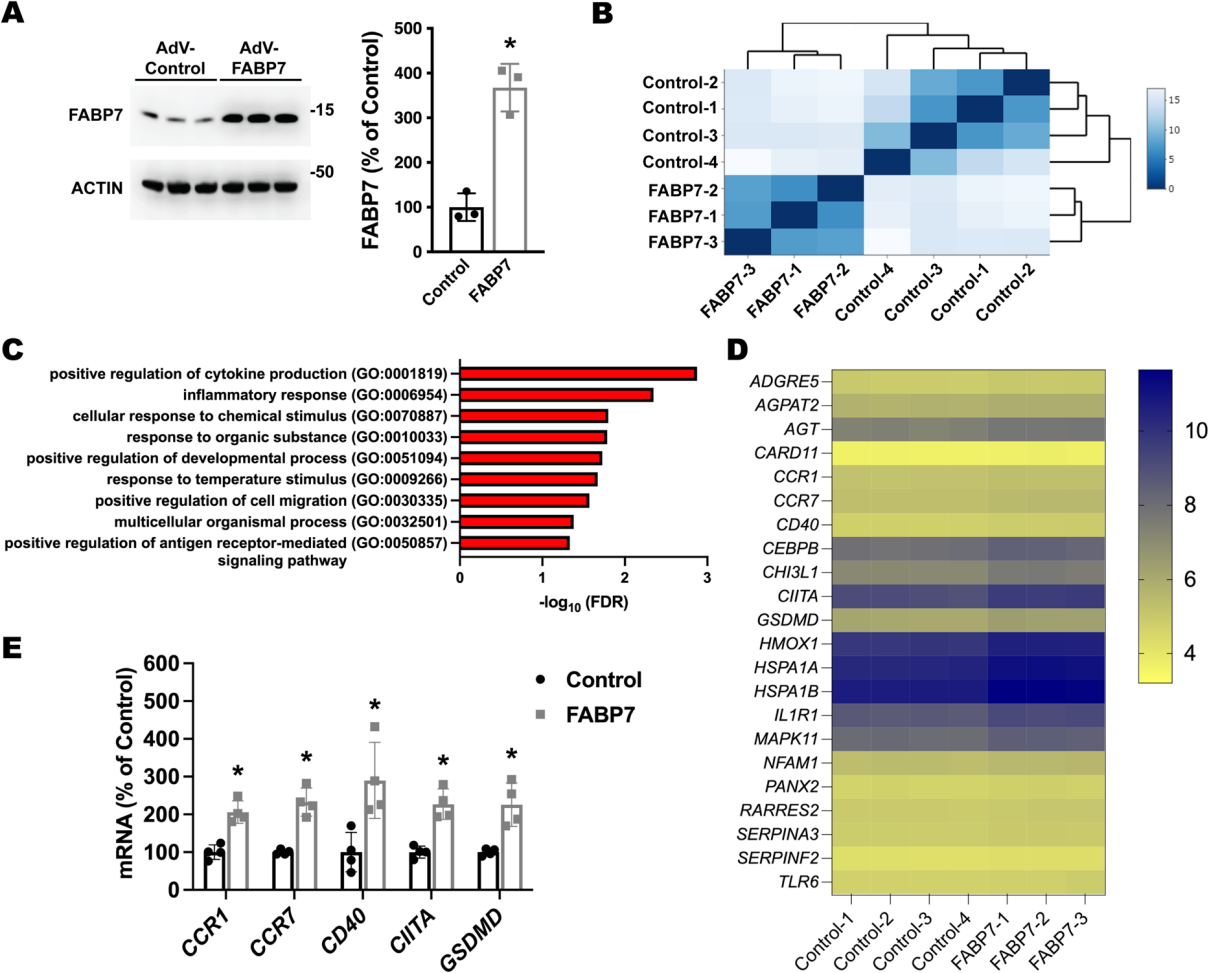
RNA-sequencing analysis of transcriptomic changes induced by FABP7 overexpression in human iPSC-derived astrocyte cultures. RNA-sequencing analysis of total RNA from i-astrocyte cultures 72 h after transduction with a CMV-null control adenovirus or an adenovirus encoding for FABP7. **A** Western blot analysis of FABP7 expression. Quantification is shown in the right panel. FABP7 expression was normalized by ACTIN levels and expressed as a percentage of CMV-null control cells (mean ± SD), *p < 0.05. **B** Heatmap of sample-to-sample Euclidean distance confirming the overall similarity among samples from each treatment group. **C** Overrepresented GO biological processes identified using PANTHER enrichment analysis of the upregulated protein-coding transcripts. Statistical significance was established at Benjamini-Hochberg false discovery rate (FDR) < 0.05. **D** Heatmap of normalized log_2_ expression counts for the transcripts corresponding to the top two statistically significant GO biological processes, i.e., positive regulation of cytokine production and inflammatory response. **E** Real-time PCR analysis of *CCR1*, *CCR7*, *CD40*, *CIITA*, and *GSDMD* mRNA expression in i-astrocyte cultures 72 h after transduction with a CMV-null control adenovirus or an adenovirus coding for FABP7. mRNA levels were corrected by ACTIN levels and expressed as a percentage of CMV-null control cells (mean ± SD), *p < 0.05

To perform an unbiased analysis of the effect of increased FABP7 expression on astrocytes, we evaluated the transcriptomic changes induced by FABP7 overexpression in cultured human astrocytes differentiated from induced pluripotent stem cells (iPSCs) obtained from healthy control subjects (i-astrocytes). Differentiation and maturation of i-astrocyte cultures were monitored by real-time PCR and immunostaining for selected markers (Supplementary Fig. 2). Western blot analysis showed that after 72 h, transduction with an FABP7-encoding adenovirus induces an approximately 3.7-fold increase in FABP7 expression when compared to i-astrocytes transduced with a CMV-null control adenovirus (Fig. 5A). FABP7 overexpression did not alter astrocyte viability (number of cells/well expressed as a percentage of the control group: 100.0 ± 15.82 in the control group versus 98.86 ± 18.22 in the FABP7-overexpressing group). RNA-sequencing analysis of i-astrocyte cultures 72 h after transduction identified a total of 19,778 unique transcripts (Supplemental Table 2). Initial hierarchical clustering of gene expression data indicated that all samples from FABP7-overexpressing i-astrocytes clustered together, as expected (Fig. 5B). Statistical analysis identified 500 transcripts with at least a 2-fold change in expression (adjusted *p* ≤ 0.05) (Supplemental Table 2). Gene Ontology (GO) enrichment analysis of the upregulated protein-coding transcripts (159 total) identified an overrepresentation of transcripts linked to 9 different GO biological processes (Fig. 5C). Among the enriched biological processes, the top two statistically significant terms were positive regulation of cytokine production and inflammatory response. A list of the identified differentially expressed transcripts corresponding to these two GO terms is shown in Fig. 5D. From this list, we confirmed by real-time PCR the upregulation of selected transcripts that have been shown to participate in astrocyte-mediated neuroinflammation and potentially play a role in AD pathology (Fig. 5E) [49–55].

**Fig. 6:**
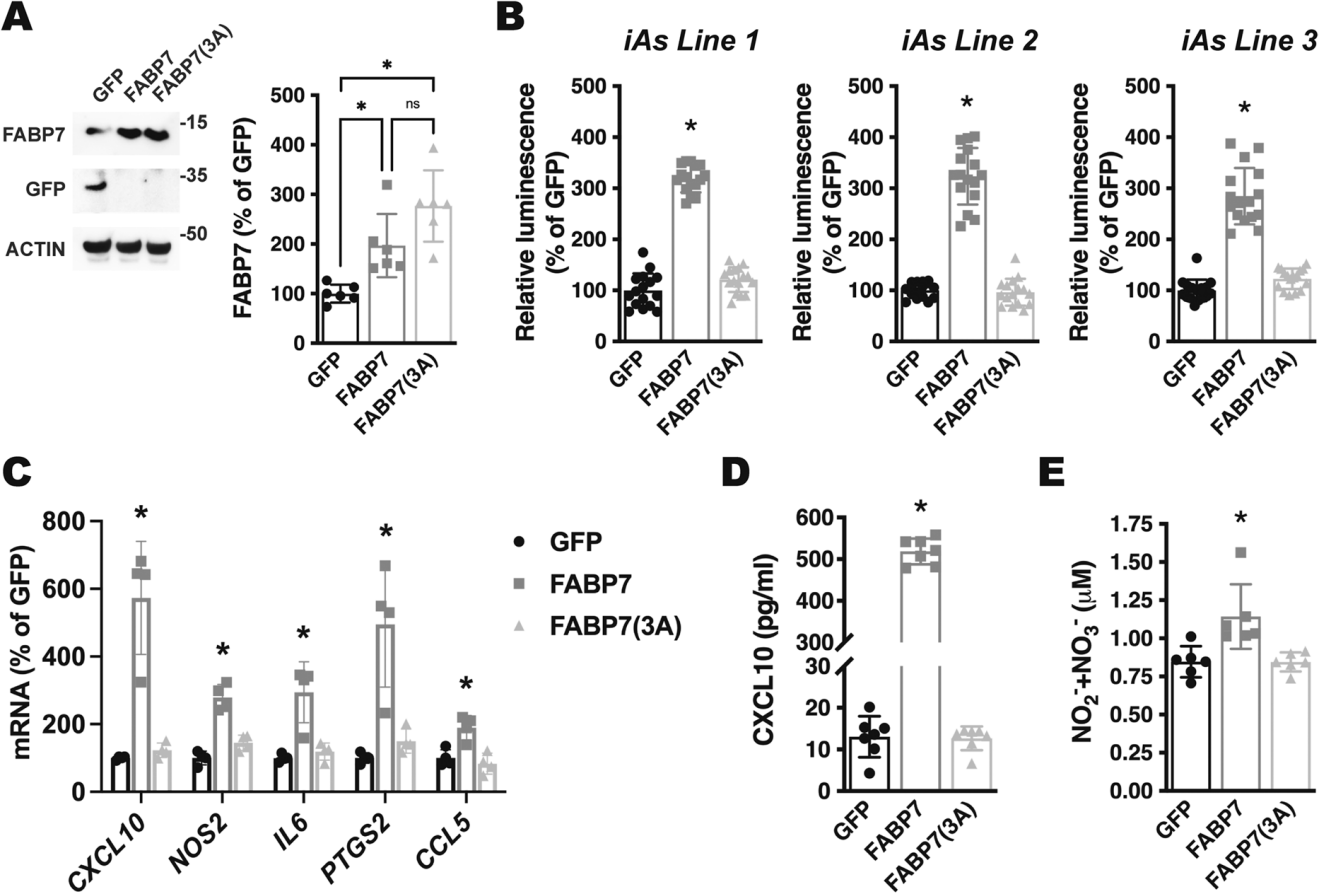
FABP7 overexpression induces an NF-κB-driven proinflammatory phenotype in human iPSC-derived astrocyte cultures. **A** Western blot analysis of FABP7 and GFP expression in line 1 i-As cultures 48 h after transduction with adenovirus coding for GFP, wild-type FABP7, or FABP7(3A). Quantification of FABP7 expression is shown in the right panel. FABP7 expression was normalized by ACTIN levels and expressed as a percentage of GFP control cells (mean ± SD), *p < 0.05. **B** Relative luminescence produced by firefly luciferase expressed under an NF-κB-driven promoter 48 h after GFP, FABP7, or FABP7(3A) overexpression in three different i-astrocyte (i-As) lines derived from healthy subjects. Confluent i-astrocyte cultures were co-transduced with a firefly luciferase construct under the control of a minimal promoter with tandem NF-κB response elements and a *Renilla* luciferase construct under a constitutive promoter. 72 h later, cultures were transduced with adenovirus expressing GFP, wild-type FABP7, or FABP7(3A). Relative firefly luciferase luminescence was corrected by *Renilla* luciferase activity and expressed as percentage of GFP control cells. **C** Real-time PCR analysis of *CXCL10*, *NOS2*, *IL6*, *PTGS2*, and *CCL5* mRNA expression 48 h after GFP, FABP7, or FABP7(3A) overexpression in line 1 i-As cultures. mRNA levels were corrected by ACTIN levels and expressed as a percentage of GFP control cells. **D** CXCL10 levels in the conditioned media from line 1 i-As cultures overexpressing GFP, FABP7, or FABP7(3A). **E** Nitrite (NO2^−^) and nitrate (NO3^−^) levels in the conditioned media from line 1 i-As cultures overexpressing GFP, FABP7, or FABP7(3A). For all panels, data are expressed as mean ± SD (*p < 0.05)

The NF-κB signaling pathway is a key regulator of cytokine production and the inflammatory response [56]. To investigate if FABP7 upregulation is capable of inducing NF-κB activation in human i-astrocytes, we analyzed the activity of an NF-κB reporter construct following FABP7 overexpression. We co-transfected confluent i-astrocyte cultures with a firefly luciferase construct under the control of a minimal promoter with tandem NF-κB response elements, together with a *Renilla* luciferase construct under the control of a constitutive promoter. After 48 h, cultures were transduced with an adenovirus coding for green fluorescent protein (GFP), wild-type FABP7, or a mutant version of FABP7 lacking ligand binding, due to the substitution of three critical residues in the ligand binding pocket [FABP7(3A); R107A/R127A/Y129A)] [31, 46, 57]. We confirmed that wild-type FABP7 and mutant FABP7(3A) were overexpressed at similar levels (Fig. 6A). Wild-type FABP7 increased the activity of the reporter, inducing an approximately 3-fold increase in firefly luciferase activity. On the other hand, overexpression of mutant FABP7(3A) did not affect the activity of the NF-κB reporter (Fig. 6B). The same results were obtained using three different lines of i-astrocytes obtained from three different healthy subjects. To further confirm the activation of NF-κB signaling after FABP7 overexpression, we used real-time PCR to assess the expression of NF-κB target genes that have been shown to modulate neuronal function and survival in neuroinflammatory or neurodegenerative conditions, including C-X-C motif chemokine ligand 10 (CXCL10), inducible nitric oxide synthase (NOS2), interleukin 6 (IL6), COX2 (PTGS2), and C-C motif chemokine ligand 5 (CCL5) [58–62]. We observed that the overexpression of wild-type FABP7, but not FABP7(3A), induces the expression of NF-κB target genes (Fig. 6C). We confirmed by ELISA that the levels of CXCL10 protein increased only in the conditioned media of i-astrocytes overexpressing wild-type FABP7 (Fig. 6D). In addition, the upregulation of *NOS2* mRNA expression was paralleled by an increase in nitric oxide synthase activity, as indicated by increased nitrite (NO2^−^) and nitrate (NO3^−^) levels in the conditioned media from wild-type FABP7-overexpressing i-astrocytes (Fig. 6E).

**Fig. 7:**
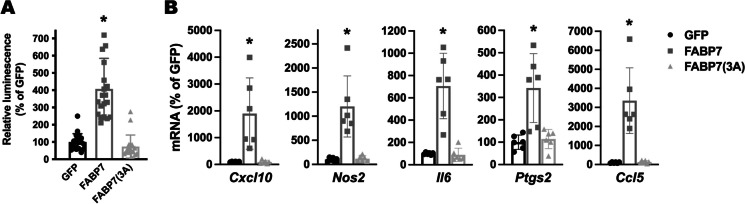
Effect of FABP7 overexpression in primary mouse hippocampal astrocytes. **A** Relative luminescence produced by firefly luciferase expressed under an NF-κB-driven promoter 48 h after GFP, FABP7, or FABP7(3A) overexpression in hippocampal astrocytes isolated from non-transgenic mice. Confluent cultures were co-transduced with an NF-κB-driven firefly luciferase construct and a *Renilla* luciferase construct under the control of a constitutive promoter. 72 h later, cultures were transduced with adenovirus expressing GFP, wild-type FABP7, or FABP7(3A). Relative firefly luciferase luminescence was corrected by the amount of *Renilla* luciferase activity and expressed as a percentage of GFP control cells. **B** Real-time PCR analysis of *Cxcl10*, *Nos2*, *Il6*, *Ptgs2*, and *Ccl5* mRNA expression 48 h after GFP, FABP7, or FABP7(3A) overexpression in primary hippocampal astrocyte cultures. mRNA levels were corrected by *Rplp0* mRNA levels and expressed as a percentage of GFP control cells. For all panels, data are expressed as mean ± SD (*p < 0.05)

**Fig. 8:**
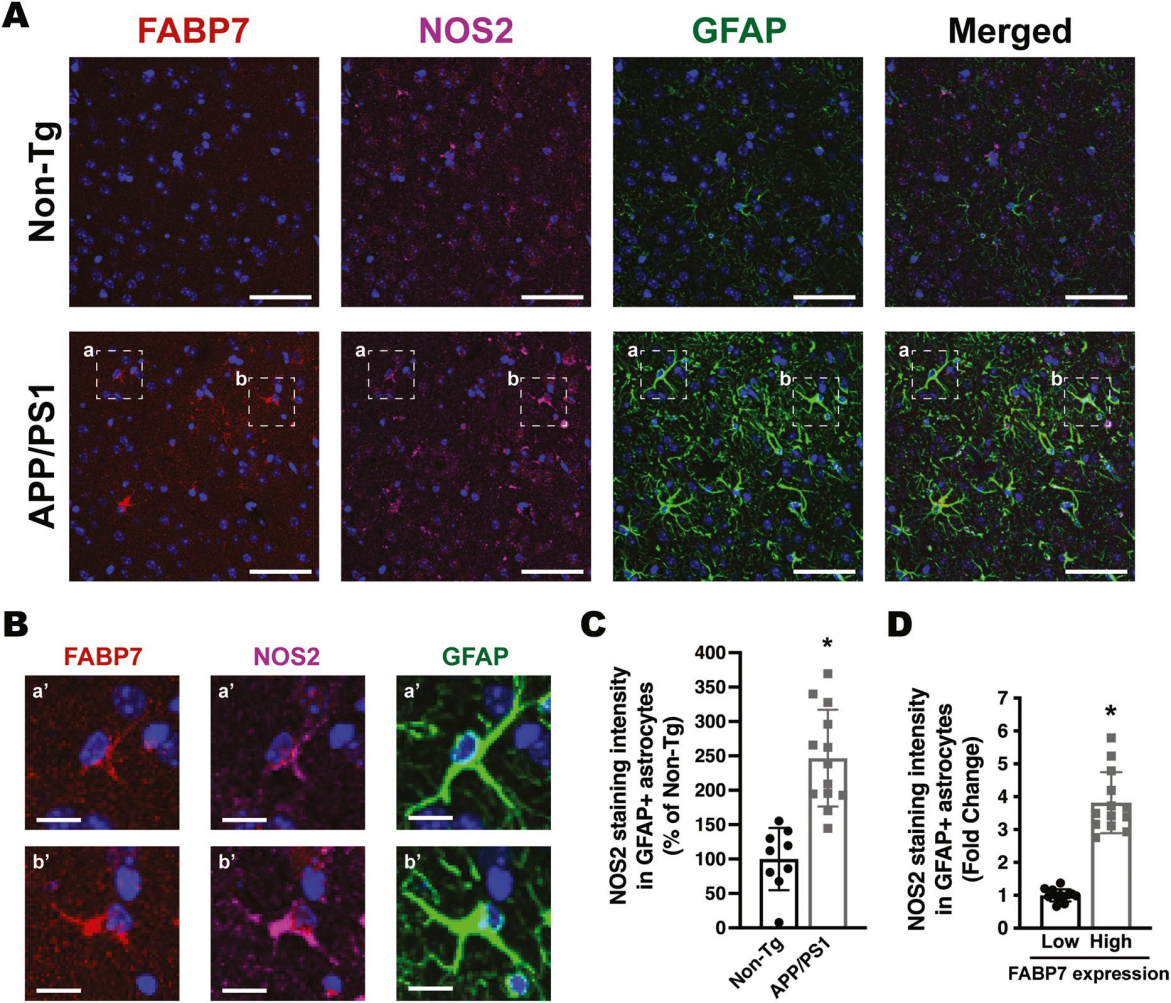
Increased NOS2 immunoreactivity in astrocytes expressing high FABP7 levels. **A** Representative maximum projection confocal microphotographs showing FABP7 (red), NOS2 (magenta), and GFAP (green) immunostaining in the cerebral cortex of 9-month-old APP/PS1 mice and age-matched non-transgenic (Non-Tg) mice. Nuclei were counterstained with DAPI (blue). Scale bar: 50 μm. **B** Higher magnification of the boxed areas (a and b) indicated in **A**. Scale bar: 10 μm. **C** Quantification of NOS2 staining intensity in GFAP+ astrocytes in the cerebral cortex of 9-month-old APP/PS1 and age-matched Non-Tg mice. **D** Quantification of NOS2 staining intensity in GFAP+ astrocytes displaying low or high levels of FABP7 expression. GFAP+ astrocytes with high FABP7 expression were defined as those having FABP7 staining intensity greater than the median observed in non-transgenic animals. For **C** and **D**, 2–3 images per animal were analyzed, *n* = 5 animals/genotype; data are expressed as mean ± SD, *p < 0.05

**Fig. 9:**
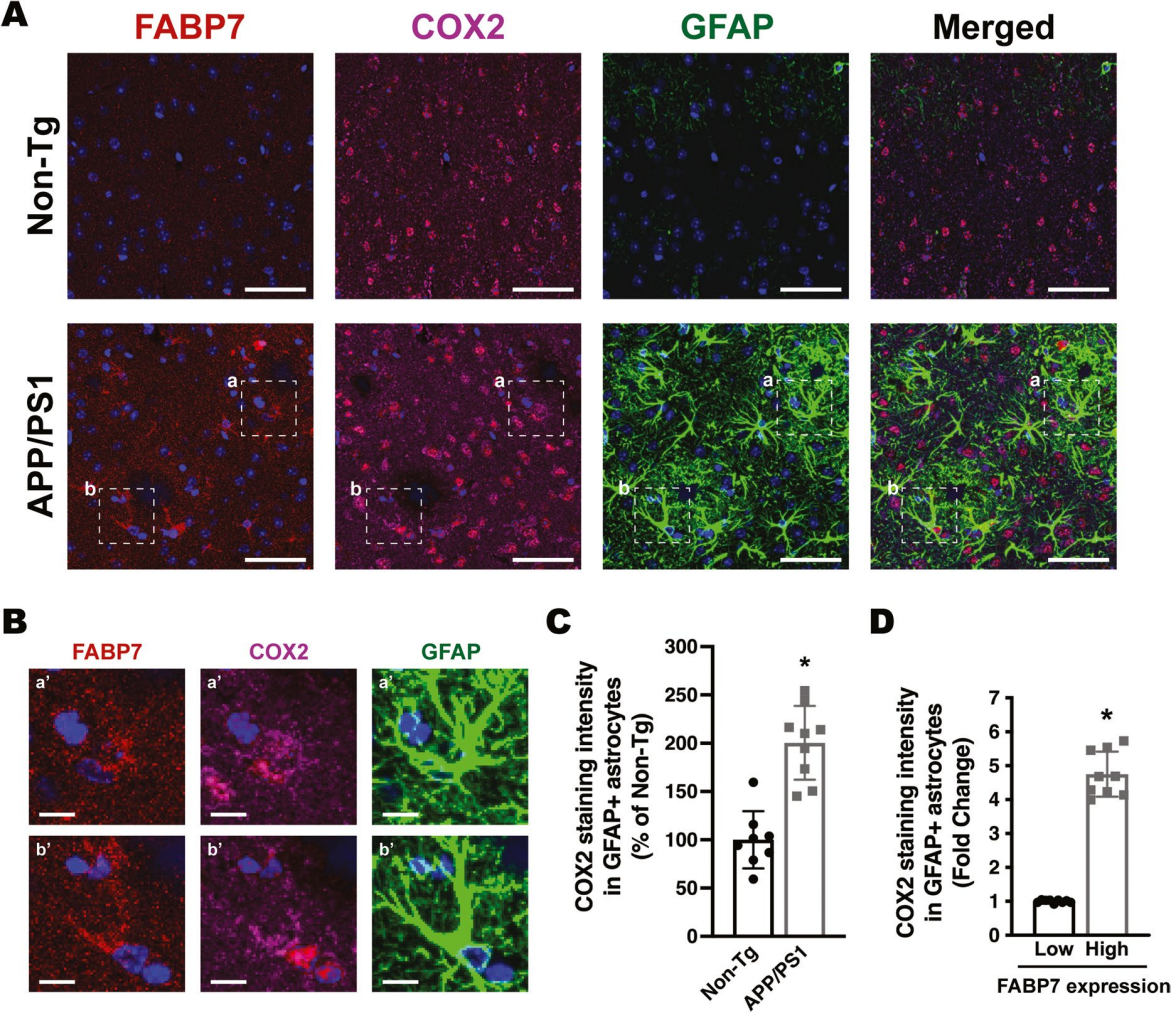
Increased COX2 immunoreactivity in astrocytes expressing high FABP7 levels. **A** Representative maximum projection confocal microphotographs showing FABP7 (red), COX2 (magenta), and GFAP (green) immunostaining in the cerebral cortex of 9-month-old APP/PS1 mice and age-matched non-transgenic (Non-Tg) mice. Nuclei were counterstained with DAPI (blue). Scale bar: 50 μm. **B** Higher magnification of the boxed areas (a and b) indicated in **A**. Scale bar: 10 μm. **C** Quantification of COX2 staining intensity in GFAP+ astrocytes in the cerebral cortex of 9-month-old APP/PS1 and age-matched Non-Tg mice. **D** Quantification of COX2 staining intensity in GFAP+ astrocytes displaying low or high levels of FABP7 expression. GFAP+ astrocytes with high FABP7 expression were defined as those having FABP7 staining intensity greater than the median observed in non-transgenic animals. For **C** and **D**, 2–3 images per animal were analyzed, *n* = 4–5 animals/genotype; data are expressed as mean ± SD, *p < 0.05

Increased NF-κB reporter activity and upregulation of NF-κB target genes were also observed in primary mouse hippocampal astrocyte cultures after FABP7 overexpression (Fig. 7). To evaluate the relevance of the in vitro results, we performed co-immunostaining of FABP7 with GFAP and NOS2 or COX2 in 9-month-old APP/PS1 or non-transgenic mice. We observed an increase in the average NOS2 and COX2 staining intensity in GFAP+ cortical astrocytes in the brain of APP/PS1 mice when compared to age-matched non-transgenic animals (Figs. 8 and 9). Moreover, astrocytes displaying high FABP7 expression (intensity of FABP7 staining greater than the median observed in non-transgenic mice) showed an increase in the average staining intensity of NOS2 (Fig. 8D) and COX2 (Fig. 9D).

## Discussion

Astrocytes are key regulators of central nervous system homeostasis and increasing evidence underscores their potential as therapeutic targets in neurodegenerative diseases [63, 64]. Here, we show that in a widely used mouse model of AD (APP/PS1 mice) and AD patients, reactive astrocytes display increased FABP7 expression. The upregulation of FABP7 expression was evident in GFAP+ astrocytes located in the vicinity of amyloid plaques (within 50 μm from the edge of a plaque), which displayed increased FABP7 staining intensity when compared to non-plaque-associated astrocytes. However, it is worth noting that the upregulation of FABP7 expression did not appear to occur in all astrocytes, evidencing the heterogeneity of reactive astrocytes in vivo. In addition, we observed that treatment of primary hippocampal astrocyte cultures with Aβ fragment 25–35 induces upregulation of FABP7 expression. This suggests that the increase in astrocytic FABP7 expression occurring in AD could be a consequence of the underlying amyloid pathology.

In astrocyte cultures, an increase in FABP7 expression leads to the development of a proinflammatory phenotype which involves increased NF-κB signaling. This effect of FABP7 in the modulation of astrocyte-mediated inflammatory response was evidenced by RNA-seq analysis of iPSC-derived human astrocyte cultures overexpressing FABP7, which identified among the upregulated targets an overrepresentation of transcripts involved in positive regulation of cytokine production and inflammatory response. An increase in NF-κB transcriptional activity following FABP7 overexpression was confirmed using a reporter assay and further evidenced by an increase in the production of proinflammatory mediators, including nitric oxide and CXCL10. This proinflammatory phenotype induced by increased FABP7 expression is observed in both human i-astrocytes and mouse primary astrocyte cultures. In the brain of 9-month-old APP/PS1 mice, cortical astrocytes display increased immunoreactivity for NOS2 and COX2, two known NF-κB targets that we observed to be upregulated following FABP7 overexpression in cultured astrocytes. Accordingly, astrocytes with higher levels of FABP7 expression displayed increased NOS2 and COX2 immunoreactivity when compared to astrocytes displaying lower FABP7 expression than the median observed in non-transgenic mice. This, together with the fact that the increase in astrocytic FABP7 expression is observed not only in the APP/PS1 mouse model but also in the brain of AD patients, supports the relevance and potential contribution of this observation to the neuroinflammatory environment observed in AD pathology.

NF-κB signaling plays a key role in the regulation of the inflammatory response in astrocytes. In the APP/PS1 AD mouse model, NF-κB inhibition reduces astrogliosis and inflammatory markers, although it increases Aβ burden [65]. While NF-κB is a key inducer of inflammation, its activation triggers a complex response that can lead to the initiation of a proapoptotic program or to the promotion of cell homeostasis, depending on the stimulus and the cellular context [66, 67]. Thus, while over-activation of NF-κB is certainly harmful during neurodegeneration, total ablation of NF-κB activity can induce detrimental effects. Consequently, new therapies aimed at restoring normal NF-κB activity may be more efficient than total NF-κB inhibition. Our results indicate that an increase in FABP7 expression activates NF-κB-dependent signaling in astrocytes and suggest that FABP7 could be a preferred pharmacological target to moderate NF-κB activation.

The fact that the overexpression of a ligand-binding impaired mutant FABP7 [FABP7(3A)] does not initiate an NF-kB-driven response in astrocytes points toward the role of endogenous ligands and FABP7 lipid-trafficking function in the modulation of the inflammatory response induced by FABP7 upregulation in astrocytes. One of the mechanisms by which FABP7 can regulate inflammation is through the regulation of PPAR signaling. By controlling the intracellular trafficking of its ligands, both endogenous and pharmacological agonists, FABPs are master regulators of ligand-dependent PPAR signaling [27, 28]. In addition to their role in metabolic regulation, PPARs also contribute to the inactivation of proinflammatory genes. Ligand-dependent transrepression appears to be the predominant mechanism by which PPARγ promotes anti-inflammatory effects by antagonizing NF-κB activation through the stabilization of a co-repressor complex [68–72]. The relevance of this mechanism in the context of AD is supported by the fact that PPARγ agonists limit neuroinflammation in AD cell culture and mouse models [73, 74]. Although FABPs selectively cooperate with PPARs to regulate transcription [27, 28, 75, 76], an increase in FABP expression above a certain threshold can also limit the activity of PPARs by creating a “sink effect,” i.e., negatively regulating the availability of ligands for PPARs [30, 77, 78]. Hence, countering FABP7 upregulation observed in pathological conditions can enhance PPAR activity. Accordingly, in mouse models, genetic ablation, or pharmacological inhibition of FABP4 protects against atherosclerosis and type 2 diabetes by a mechanism involving PPARγ activation, which antagonizes NF-κB-dependent cytokine production [30, 79, 80]. A similar mechanism has been proposed for the protective effect of FABP5 inhibition in models of experimental autoimmune encephalomyelitis (EAE) [81, 82]. Likewise, the expression of FABP7 in glioblastoma cells promotes cell migration by a mechanism involving increased expression of inflammatory markers and downregulation of PPARγ signaling [31]. This suggests that the activation of NF-κB signaling observed in astrocytes after FABP7 overexpression could be mediated, at least in part, by the effects of FABP7 on PPARγ signaling.

FABP7 also contributes to regulating the production of lipid mediators by cyclooxygenases (COXs; prostaglandin G/H synthases), which are central players in the inflammatory response [31, 83]. FABPs regulate the intracellular trafficking of fatty acids to different subcellular compartments. FABP7 binds preferentially to ω-3 polyunsaturated fatty acids (PUFAs), including ω-3-eicosapentaenoic acid (EPA) and ω-3-docosahexaenoic acid (DHA), and binds ω-6-arachidonic acid (AA) with lower affinity (Kd ≈ 200 nM) [46]. After binding, FABP7 translocates the fatty acids to different intracellular compartments, including the nucleus (for regulation of PPAR signaling) and the luminal surface of the endoplasmic reticulum (ER), for metabolism by COXs [83]. In glioblastoma cells, FABP7 coordinates the utilization of AA and DHA depending on their relative availability, with an increase in the AA:DHA ratio promoting AA metabolism by COX2 [31, 83]. Remarkably, a decrease in DHA abundance in blood and brain correlates with the development of cognitive decline in aging and AD, while high dietary and plasma DHA levels decrease the risk of dementia and AD [84–87]. Thus, the upregulation of FABP7 in astrocytes, together with the higher relative availability of AA (high AA:DHA ratio), could favor the production of AA-derived proinflammatory mediators by COX2 and contribute to the establishment of chronic inflammation.

In summary, here we show that an increase in FABP7 expression is sufficient to induce a proinflammatory phenotype in human astrocytes. An upregulation in astrocytic FABP7 expression is observed in the brains of AD patients and in an AD mouse model, suggesting its potential relevance in the context of the disease. Moreover, as discussed above, FABP7 regulates PPAR signaling and the production of inflammatory mediators by COXs, two pathways that were independently considered potential targets for AD treatment [8, 9]. Together, our results suggest the potential of FABP7 as a therapeutic target to prevent the establishment of an exacerbated inflammatory response in the context of AD.

### Supplementary information

ESM 1 (PDF 37 kb)

ESM 2 (XLS 5157 kb)

ESM 3 (TIF 9.48 MB)

ESM 4 (TIF 9.40 MB)

## Data Availability

The data that support the findings of this study are available from the corresponding author upon reasonable request.
