# Supplementary material for: FABP7 drives an inflammatory response in human astrocytes and is upregulated in Alzheimer’s disease
Source: GeroScience. 2023 Sep 9;46(2):1607–25. doi: 10.1007/s11357-023-00916-0 (PMC10828232; doi:10.1007/s11357-023-00916-0)
Supplement: Supplementary file 1 — (PDF 37 kb) [file 11357_2023_916_MOESM1_ESM.pdf]

Supplemental table 1. Sequence of primers used for real-time PCR.

Target gene	***Forward (sequence 5'-3')***	***Reverse (sequence 5'-3')***
Mouse *Ccl5*	CCAGAGAAGAAGTGGGTTCAAG	AGCAATGACAGGGAAGCTATAC
Mouse *Cxcl10*	TCAGGCTCGTCAGTTCTAAGT	CCTTGGGAAGATGGTGGTTAAG
Mouse *Il6*	TTTCCTCTGGTCTTCTGGAGTA	CTCTGAAGGACTCTGGCTTTG
Mouse *Nos2*	AGGAGGAGAGAGATCCGATTTAG	TCAGACTTCCCTGTCTCAGTAG
Mouse *Ptgs2*	CGGACTGGATTCTATGGTGAAA	CTTGAAGTGGGTCAGGATGTAG
Mouse *Rplp0*	CCTCCTTCTTCCAGGCTTTG	CCACCTTGTCTCCAGTCTTTATC
Human *ACTIN*	GGATCAGCAAGCAGGAGTATG	AGAAAGGGTGTAACGCAACTAA
Human *CCL5*	TGCCCACATCAAGGAGTATTT	GATGTACTCCCGAACCCATTT
Human *CCR1*	GACTATGACACGACCACAGAGT	CCAACCAGGCCAATGACAAATA
Human *CCR7*	TGAGGTCACGGACGATTACAT	GTAGGCCCACGAAACAAATGAT
Human *CD40*	TTGGGGTCAAGCAGATTGCTA	GCAGATGACACATTGGAGAAGA
Human *CIITA*	CCTGGAGCTTCTTAACAGCGA	TGTGTCGGGTTCTGAGTAGAG
Human *CXCL10*	GTAATAACTCTACCCTGGCACTATAA	GATGGGAAAGGTGAGGGAAATA
Human *GSDMD*	GTGTGTCAACCTGTCTATCAAGG	CATGGCATCGTAGAAGTGGAAG
Human *IL6*	GGAGACTTGCCTGGTGAAA	CTGGCTTGTTCCTCACTACTC
Human *NOS2*	GTCAGAGTCACCATCCTCTTTG	GCAGCTCAGCCTGTACTTATC
Human *PTGS2*	TACTGGAAGCCAAGCACTTT	GGACAGCCCTTCACGTTATT
