# Supplementary figures and images for: FABP7 drives an inflammatory response in human astrocytes and is upregulated in Alzheimer’s disease
Source: GeroScience. 2023 Sep 9;46(2):1607–25. doi: 10.1007/s11357-023-00916-0 (PMC10828232; doi:10.1007/s11357-023-00916-0)

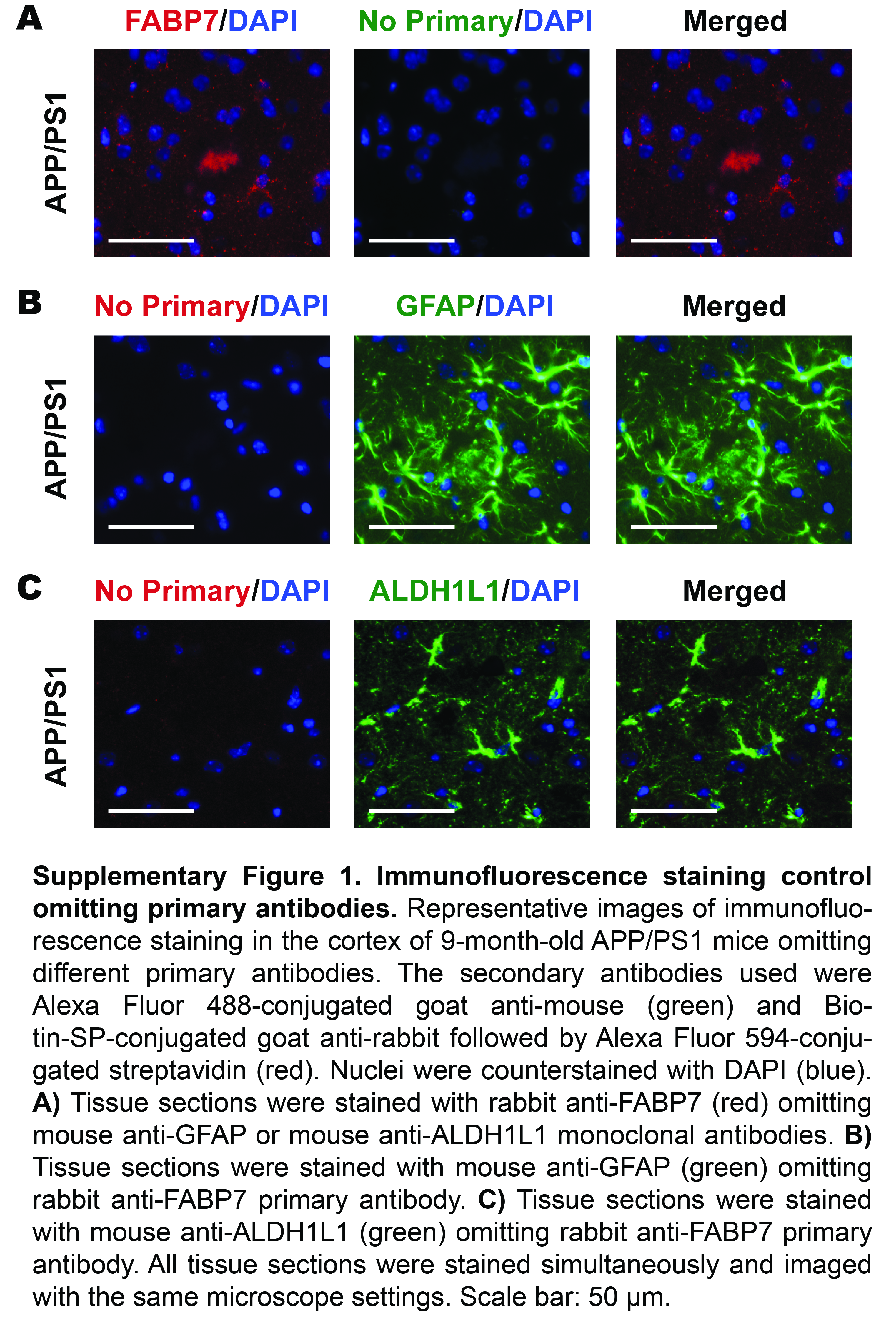

Supplement: Supplementary file 3 — (TIF 9.48 MB) [file 11357_2023_916_MOESM3_ESM.tif]

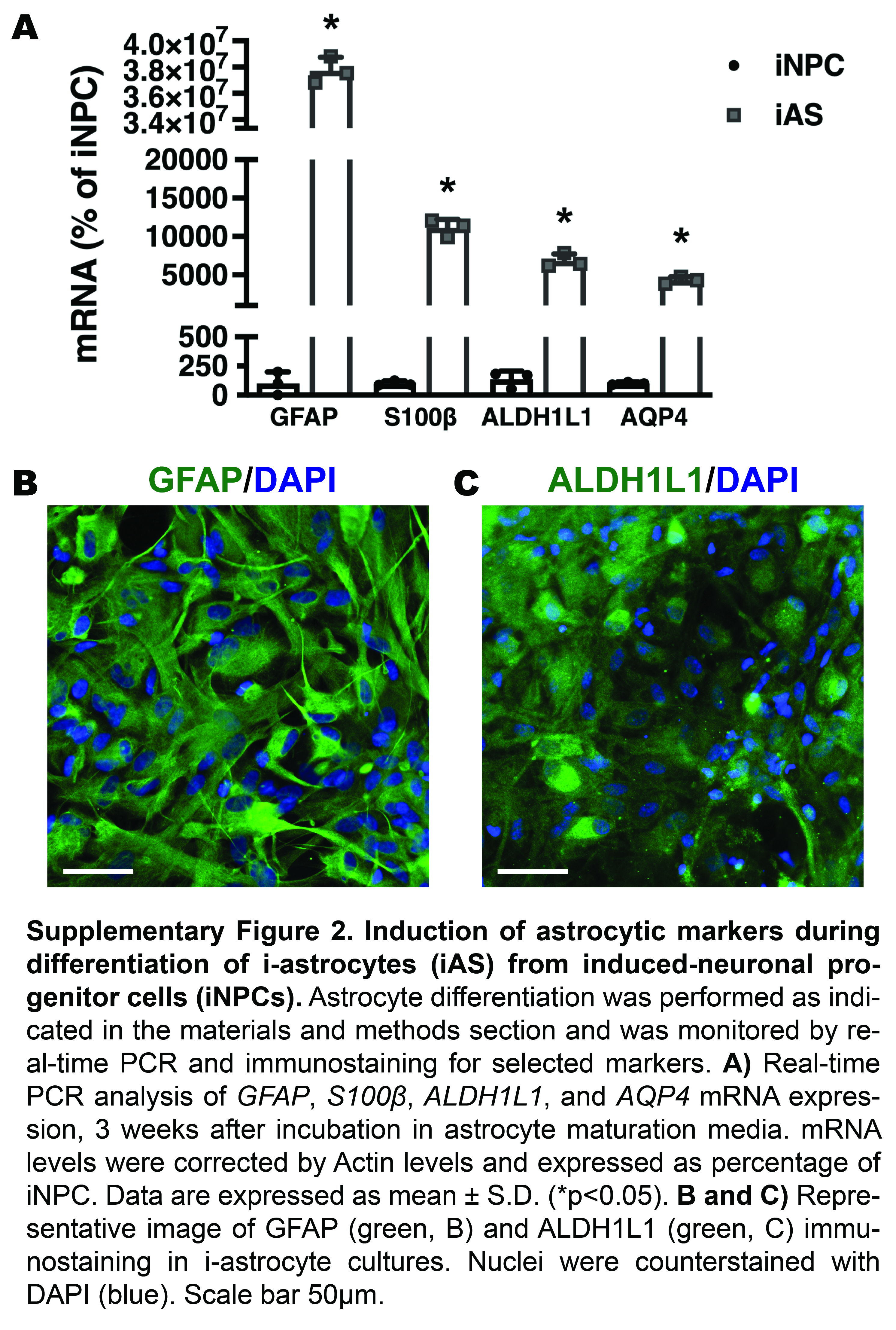

Supplement: Supplementary file 4 — (TIF 9.40 MB) [file 11357_2023_916_MOESM4_ESM.tif]
